# Supplementary material for: Recognition of Davidsoniella virescens on Fagus sylvatica Wood in Poland and Assessment of Its Pathogenicity
Source: J Fungi (Basel). 2024 Jun 29;10(7):465. doi: 10.3390/jof10070465 (PMC11278147; doi:10.3390/jof10070465)
Supplement: Supplementary file 1 [file jof-10-00465-s001.zip › jof-3052786-supplementary.pdf]

18S

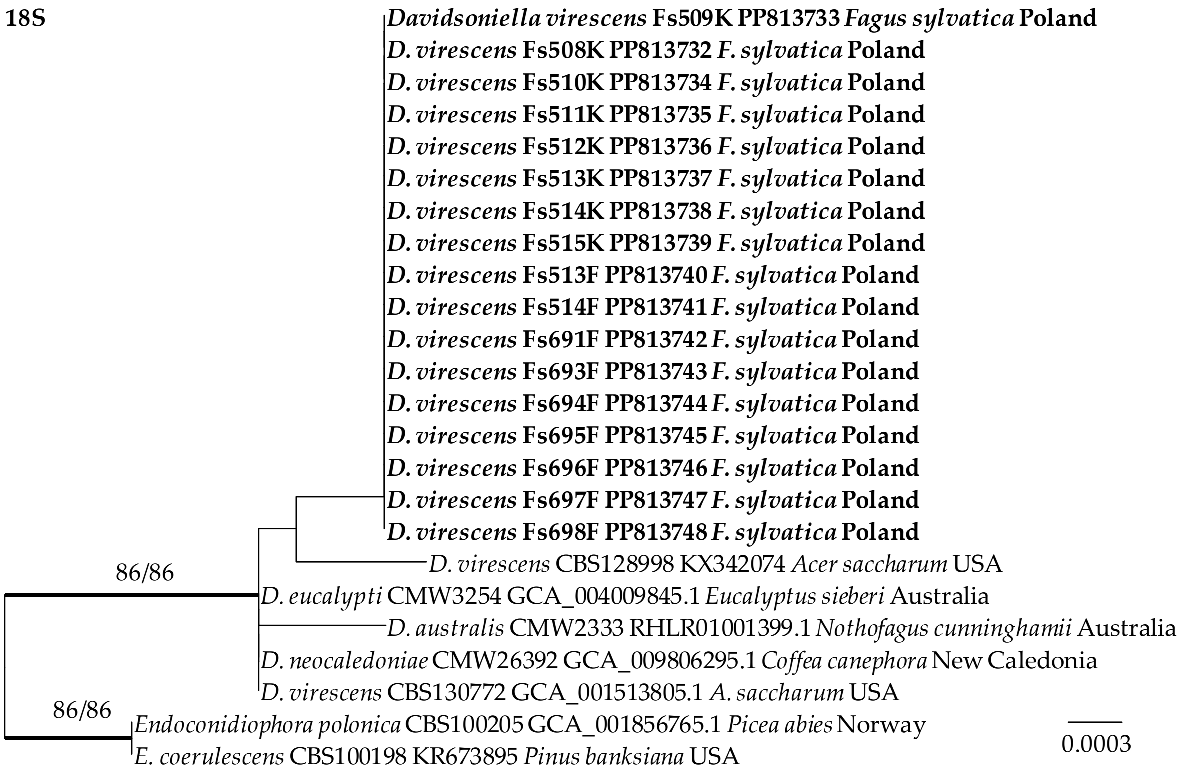

Figure S1. Phylogram obtained from analyses of small subunit (SSU) ribosomal DNA (18S) data for the genus *Davidsoniella*. Sequences obtained during this study are presented in bold type. The presented phylogram was obtained from Maximum Likelihood (ML) analyses. The bootstrap values ≥ 75% for ML and Maximum Parsimony (MP) analyses are presented at nodes as follows: ML/MP. Bold branches indicate posterior probability values ≥ 0.95 obtained from Bayesian Inference (BI) analyses. * Bootstrap values <75%. The tree is drawn to scale (see bar) with branch length measured in the number of substitutions per site. *Endoconidiophora coerulescens* and *E. polonica* represent the outgroup.

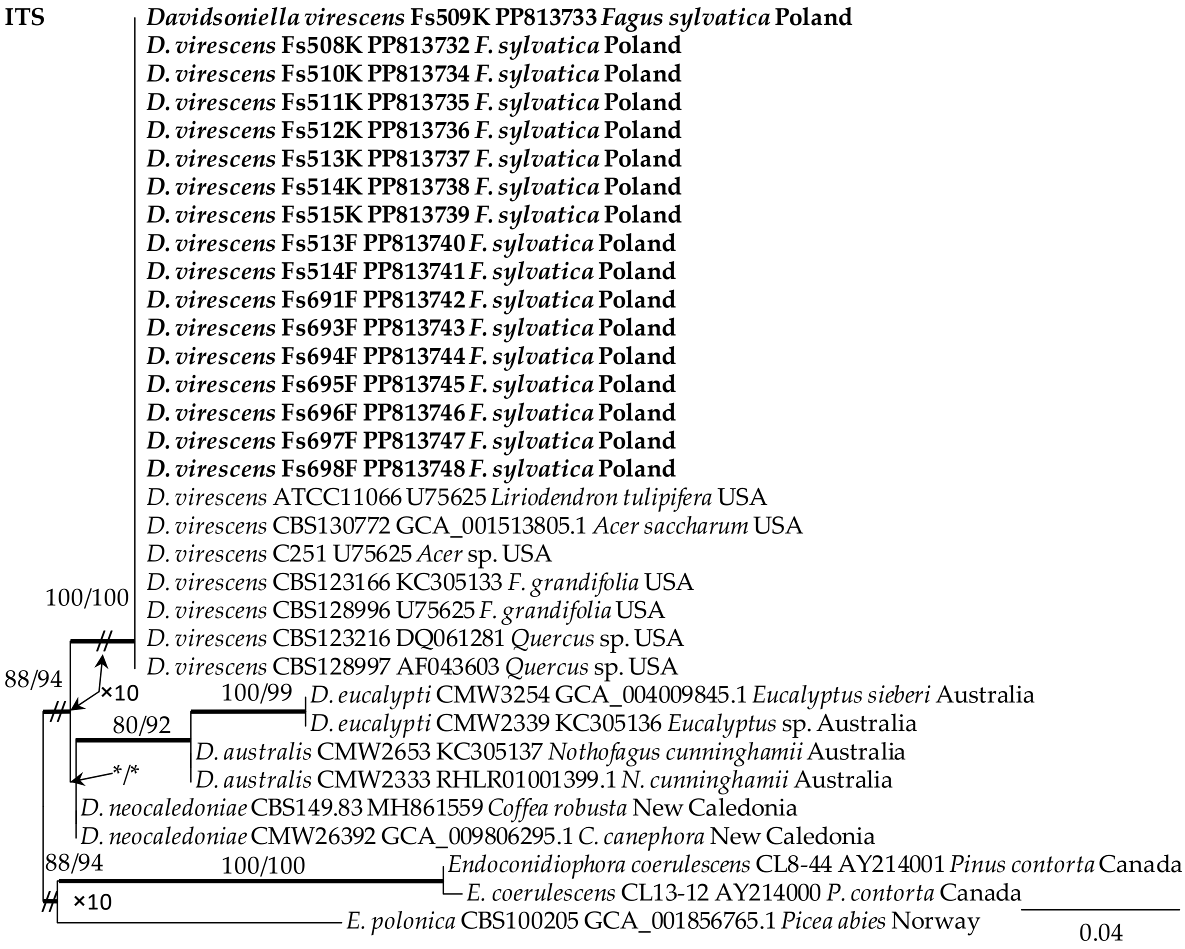

Figure S2. Phylogram obtained from analyses of internal transcribed spacer (ITS) data for the genus *Davidsoniella*. Sequences obtained during this study are presented in bold type. The presented phylogram was obtained from Maximum Likelihood (ML) analyses. The bootstrap values ≥ 75% for ML and Maximum Parsimony (MP) analyses are presented at nodes as follows: ML/MP. Bold branches indicate posterior probability values ≥ 0.95 obtained from Bayesian Inference (BI) analyses. * Bootstrap values <75%. The tree is drawn to scale (see bar) with branch length measured in the number of substitutions per site. *Endoconidiophora coerulescens* and *E. polonica* represent the outgroup.

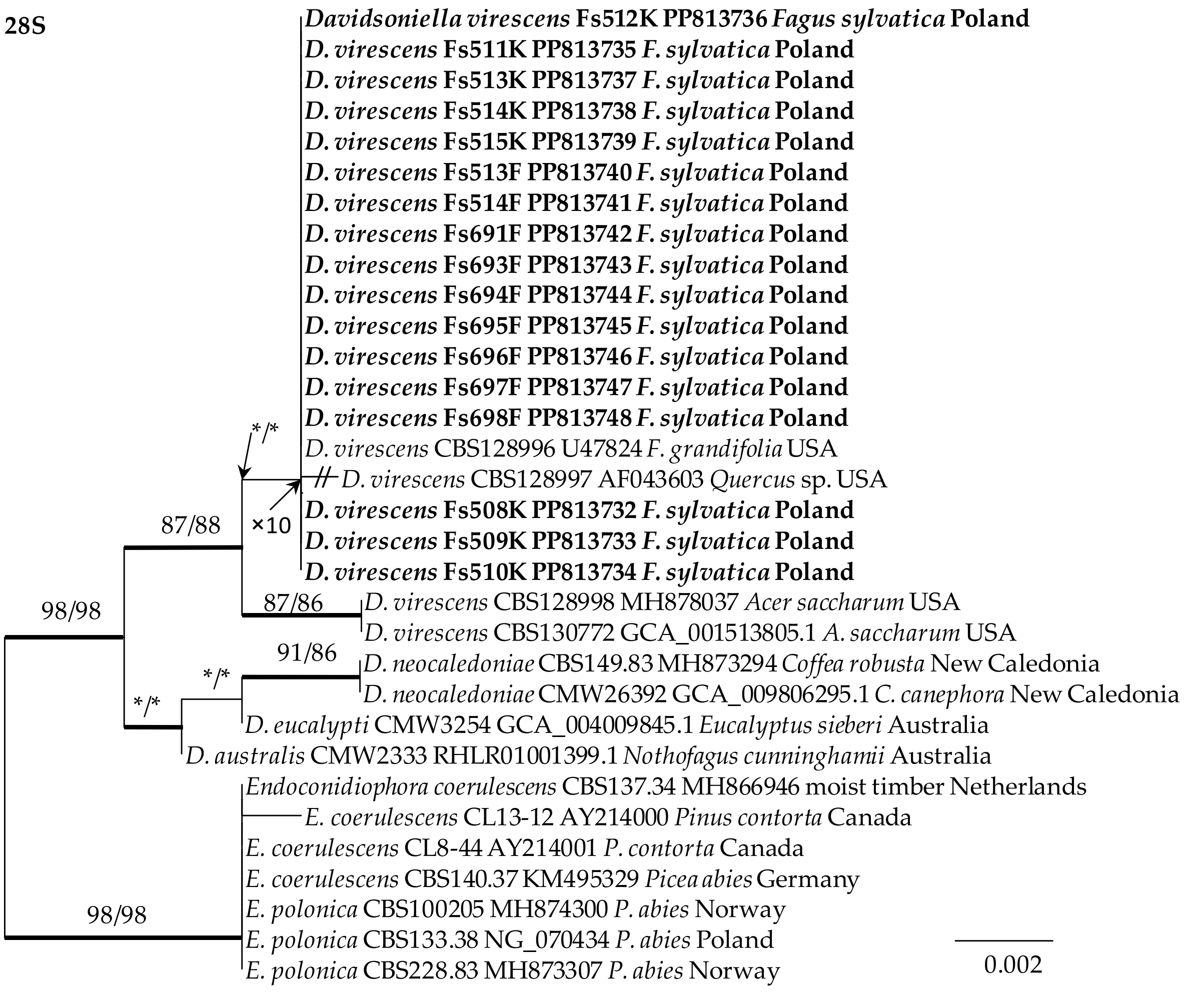

Figure S3. Phylogram obtained from analyses of large subunit (LSU) ribosomal DNA (28S) data for the genus *Davidsoniella*. Sequences obtained during this study are presented in bold type. The presented phylogram was obtained from Maximum Likelihood (ML) analyses. The bootstrap values ≥ 75% for ML and Maximum Parsimony (MP) analyses are presented at nodes as follows: ML/MP. Bold branches indicate posterior probability values ≥ 0.95 obtained from Bayesian Inference (BI) analyses. * Bootstrap values <75%. The tree is drawn to scale (see bar) with branch length measured in the number of substitutions per site. *Endoconidiophora coerulescens* and *E. polonica* represent the outgroup.

Table S1. Primers used in this study to amplify and sequence barcode regions in molecular identification.

Gene	Primer	Sequence	References
β-tubulin 2 (TUB2)	T10PBCer	5′-TCTATAGGTCCACCTCCAGAC-3′	This study
	Bt2b	5′-ACCCTCAGTGTAGTGACCCTTGGC-3′	Glass and Donaldson [38]
The small subunit (18S) of the nuclear rDNA (SSU)	SSJ	5′-CTGGTTGATCCTGCCAGTAG-3′	Hausner et al. [39]
	SST	5′-ACGGAGACCTTGTTACGACT-3′	Hausner et al. [39]
Internal transcribed spacer (ITS)	ITS5	5′-GGAAGTAAAAGTCGTAACAAGG-3′	White et al. [40]
	ITS4	5′-TCCTCCGCTTATTGATATGC-3′	White et al. [40]
The large subunit (28S) of the nuclear rDNA (LSU)	LR0R	5′-ACCCGCTGAACTTAAGC-3′	Rehner and Samuels [41]
	LR6	5′-CGCCAGTTCTGCTTACC-3′	Vilgalys and Hester [42]
Translation elongation factor 1-α (TEF1)	EFCF1	5′-AGTGCGGTGGTATCGACAAG-3′	Oliveira et al. [43]
	EFCF2	5′-TGCTCACGGGTCTGGCCAT-3′	Oliveira et al. [43]
	EFCF3	5′-ATGGCCAGACCCGTGAGCA-3′	Oliveira et al. [43]
	EFCF6PB [1]	5′-CATRTCACGGACGGCGAARC-3′	This study

[1] Degenerate primer.

Table S2. Isolates and GenBank accession numbers for reference sequences used in the phylogenetic analyses.

Species	Strains	Host	Collector	Origin	GenBank Accession Numbers				
					18S	ITS	28S	TUB2	TEF1
Davidsoniella australis	CMW 2333	*Nothofagus cunninghamii*	M. Hall	Australia	RHLR01001399	RHLR01001399	RHLR01001399	RHLR01001399	RHLR01001399
	CMW 2653	*Nothofagus cunninghamii*	?	Australia	–	KC305137	–	–	–
	C 631	*Nothofagus cunninghamii*	Glen A. Kile	Australia	–		–	–	HM569648
	CMW 2339	*Eucalyptus* sp.	?	Australia	–	KC305136	–	KC336012	–
	CMW 3254^{E}	*Eucalyptus sieberi*	Mark J. Dudzinski	Australia	GCA_004009845	GCA_004009845	GCA_004009845	GCA_004009845	HM569647
	CMW 26392	*Coffea canephora*	?	New Caledonia	GCA_009806295	GCA_009806295	GCA_009806295	GCA_009806295	GCA_009806295
	CBS 149.83^{T}	*Coffea robusta*	Roger Dadant	New Caledonia	–	MH861559	MH873294	–	HM569649
	ATCC11066^{T}	*Liriodendron tulipifera*	George Henry Hepting	USA	–	U75625	–	–	–
	CBS 130772	*Acer saccharum*	David Houston	USA	GCA_001513805	GCA_001513805	GCA_001513805	GCA_001513805	GCA_001513805
	C 251	*Acer* sp.	David Houston	USA	–	U75625	–	–	–
	CBS 128998	*Acer saccharum*	David Houston	USA	KX342074	–	MH878037	–	HM569645
	CBS 123166	*Fagus grandifolia*	David Houston	USA	–	KC305133		KC336011	EF070413
	CBS 128996	*Fagus grandifolia*	David Houston	USA	–	U75625	U47824	–	HM569646
	CBS 123216	*Quercus* sp.	Thomas Hinds	USA	–	DQ061281	–	–	AY529011

	CBS 128997	*Quercus* sp.	Thomas Hinds	USA	–	AF043603	AF043603	–	–
Endoconidiophora coerulescens	CBS 100198	*Pinus banksiana*	Thomas Harrington	USA	KR673895	–	–	–	HM569653
	CL8-44	*Pinus contorta*	Peter Arne Loppnau	Canada	–	AY214001	AY214001	–	–
	CL13-12	*Pinus contorta*	Peter Arne Loppnau	Canada	–	AY214000	AY214000	–	–
	CBS 137.34	Moist timber	Kneteman	Netherlands	–	–	MH866946	–	–
	CBS 140.37	*Picea abies*	T. Rohde	Germany	–	–	KM495329	–	–
Endoconidiophora polonica	CBS 100205[N]	*Picea abies*	Halvor Solheim	Norway	GCA_001856765	GCA_001856765	MH874300	GCA_001856765	HM569656
	CBS 133.38	*Picea abies*	Wincenty Siemaszko	Poland	–	–	NG_070434	–	–
	CBS 228.83	*Picea abies*	Halvor Solheim	Norway	–	–	MH873307	–	–

[T] Type, [E] Ex-holotype, [N] Ex-neotype.
